# Cellular and molecular aspects of adult brain development in honeybee castes (*Apis mellifera L*.)

**DOI:** 10.1186/1753-6561-8-S4-P159

**Published:** 2014-10-01

**Authors:** Aline Vomero, Heloisa Gianelli, Marcio T Oliveira, Maíra B Lima, Pedro HZ Vitoria, Paulo E Alvarenga, Lívia MR Moda, Joseana Vieira, Ana Bomtorin, Zilá LP Simões, Angel Roberto Barchuk

**Affiliations:** 1 Laboratory of Integrative Animal Biology, Federal University of Alfenas, MG, Brazil; 2 Laboratory of Developmental Biology of Honeybees, São Paulo State University, São Paulo, SP, Brazil

## Background

The adult honey bee brain exhibits a complex architecture composed by millions of neurons, glial cells and their respective tracts which form structures known as neuropils. They are organized to produce the optic lobe, antennal lobe, central complex and mushroom bodies. Learning and memory-related skills that honeybee workers use for navigation, foraging, nestmate recognition, and other activities are believed to be associated with the mushroom bodies, which are more developed in the adult members of the worker caste. During larval period, however, the differential feeding offered to prospective queens promotes faster brain development and higher expression of several neurogenic genes (*ataxin*-*2, cryptocephal, dachshund, Eph Receptor, fax, shot, krüppel homolog-1 *and *tetraspanin 5D*) [1]. It seems that in some point during pupation, there happens a shift in this trend. In fact, queen's brain experiences extensive cell death events, but worker's brain is favored by higher rates of cell proliferation, resulting in caste specific brains [2].

## Methods

*A. mellifera *pupae were collected from colonies (africanized hybrids) at the Experimental Apiary of the Federal Institute of Muzambinho (IFSULDEMINAS) at Muzambinho, Minas Gerais State, Brazil. The developmental stages were classified according to the criteria proposed by Michelette and Soares [3] and Rembold *et al*[4]. To investigate whether differences could be detected in brain development in the context of caste differentiation we performed neuroanatomical studies of whole mount preparations of queens and workers pupal brains using actin phalloidin/rhodamin to visualize axons, and DAPI to visualize nuclei. In addition, we used genome-wide expression analyses and normalized transcript expression experiments using RT-qPCR for monitoring specific genes during pupal stages.

## Results and conclusions

Our morphological results showed that during early pupal stages there are no obvious differences between castes brain development. However, late worker pupae exhibit larger calyces, fundamental parts of mushroom bodies, when compared with queens. Gene expression analyses revealed that the transcription profiles in brains of queens and workers (except for *kr-h1*) show an opposite pattern to that observed during larval development, with workers' brain with higher levels of gene transcription (e.g., *mesencephalic astrocyte derived neurotrophic factor, minibrain, signal peptide peptidase*, and *tumbleweed*, all associated to neurogenic events or cell death prevention). This indicates that the respective protein products are responsible for the differential development of adult brains between honeybee castes. Particularly, the expression patterns of the neurogenic genes *atx-2 *and *fax *and the "anti-cell death" gene *signal peptide peptidase *help explain the observed inversion of the neuroblasts area in queens and workers' brain, which, during larval period favors queens (very likely due to the differential feeding), and during pupal development and in adults, favors workers.
